# A needs assessment of resuscitative endovascular balloon occlusion of the aorta (REBOA) in non-traumatic out-of-hospital cardiac arrest in Norway

**DOI:** 10.1186/s12873-020-00324-z

**Published:** 2020-04-21

**Authors:** Jostein Rødseth Brede, Jo Kramer-Johansen, Marius Rehn

**Affiliations:** 1 Department of Emergency Medicine and Pre-Hospital Services, St. Olav University Hospital, Trondheim, Norway; 2 Norwegian Air Ambulance Foundation, Department of Research and Development, Oslo, Norway; 3 Department of Anesthesiology and Intensive Care Medicine, St. Olav’s University Hospital, Prinsesse Kristinas Gate 3, 7030 Trondheim, Norway; 4 Department of Circulation and MedicalImaging, Faculty of Medicine and Health Sciences, Norwegian University of Science and Technology (NTNU), Trondheim, Norway; 5 Division of Prehospital Services, Air Ambulance Department, Oslo University Hospital, Oslo, Norway; 6 Norwegian National Advisory Unit for Prehospital Emergency Care (NAKOS), Oslo, Norway; 7 Institute of Clinical Medicine, Faculty of Medicine, University of Oslo, Oslo, Norway; 8 Faculty of Health Sciences, University of Stavanger, Stavanger, Norway

**Keywords:** Aortic occlusion, Cardiac arrest, Cardiopulmonary resuscitation, REBOA

## Abstract

**Introduction:**

Out of hospital cardiac arrest (OHCA) carries an 86% mortality rate in Norway. Resuscitative endovascular balloon occlusion of the aorta (REBOA) is a potential adjunct in management of non-traumatic cardiac arrest and is feasible in pre-hospital setting without compromising standard cardiopulmonary resuscitation (CPR). However, number of patients potentially eligible for REBOA remain unknown. In preparation for a clinical trial to investigate any benefit of pre-hospital REBOA, we sought to assess the need for REBOA in Norway as an adjunct treatment in OHCA.

**Methods:**

Retrospective observational cohort study of data from the Norwegian Cardiac Arrest Registry in the 3-year period 2016–2018. We identified number of patients potentially eligible for pre-hospital REBOA during CPR, defined by suspected non-traumatic origin, age 18–75 years, witnessed arrest, ambulance response time less than 15 min, treated by ambulance personnel and resuscitation effort over 30 min.

**Results:**

In the 3-year period, ambulance personnel resuscitated 8339 cases. Of these, a group of 720 patients (8.6%) were eligible for REBOA. Only 18% in this group achieved return of spontaneous circulation and 7% survived for 30 days or more.

**Conclusion:**

This national registry data analysis constitutes a needs assessment of REBOA in OHCA. We found that each year approximately 240 patients, or nearly 9% of ambulance treated OHCA, in Norway is potentially eligible for pre-hospital REBOA as an adjunct treatment to standard resuscitation. This needs assessment suggests that there is sufficient patient population in Norway to study REBOA as an adjunct treatment in OHCA.

## Background

Of all patients treated for out of hospital cardiac arrest (OHCA) in Norway, one third regains spontaneous circulation (ROSC), but overall, 86% don’t survive [1]. Oxygen delivery to the brain and heart are pivotal and maintenance of circulation with cardio-pulmonary resuscitation (CPR) is beneficial, but often futile if the precipitating cause of arrest cannot be identified and remedied. The number of OHCA in Norway was 3172 in 2017, which correlates to an OHCA incidence of 60/100000 inhabitants per year, increasing from 53/100000 in 2015 [1]. Many patients with OHCA carry little comorbidity with potential for good long-term functional recovery [2].

Resuscitative endovascular balloon occlusion of the aorta (REBOA) can be applied in management of haemorrhagic shock or cardiac arrest (CA) secondary to trauma. Further, REBOA has been advocated as an adjunct in management of non-traumatic cardiac arrest patients [3, 4]. Animal studies show that REBOA during cardiopulmonary resuscitation (CPR) provide both increased coronary artery blood flow and perfusion pressure and increased rates of return of spontaneous circulation (ROSC) [5–11]. In humans, increased coronary perfusion pressure is associated with ROSC [12]. REBOA during experimental CPR also increase blood flow to the carotids [8, 13], and cerebral arteries [6, 7, 13–15] with subsequent increased cerebral perfusion pressure [6, 7, 13, 16]. Hence, patients with non-traumatic CA might benefit from REBOA during CPR. Currently, only one study reports the prospective use of REBOA in clinical use [17]. This study demonstrates that pre-hospital REBOA procedure during resuscitation is feasible and does not influence advanced cardiovascular life support (ACLS).

Several studies estimate number of patients with potential benefit from REBOA in treatment of haemorrhagic trauma [18–20]. However, no study has yet reported number of cardiac arrest patients that potentially may benefit from pre-hospital REBOA. Whether REBOA or any other invasive procedure applied late in resuscitation scenario, improves outcome for OHCA patients remain unknown but should be investigated in clinical trials. In preparation for such a trial, we sought to assess the number of patients eligible for REBOA as an adjunct treatment in OHCA, with the use of data from the Norwegian Cardiac Arrest Registry (NORCAR).

## Methods

This is a retrospective observational cohort study following the STROBE guideline [21], on adult patient data (age 18–75 years) captured in NORCAR in the 3-year period January 1st 2016 to December 31st 2018. NORCAR is a mandatory national health registry, hosted by Oslo University Hospital. The registry aim to monitor and improve healthcare provided to people with cardiac arrest. The registry is a resuscitation registry and all patients treated by bystanders or health care professionals with CPR or defibrillation are included. All Norwegian health trusts reports OHCA to this registry into a central database (Medical Registry System) that ensures data integrity, privacy and security [1]. NORCAR captures data based on a modified Utstein template on patient and event characteristics, as well as treatment and outcomes grouped by Emergency Medical Communications Centre (EMCC), ambulance services, and hospitals.

Centrality index reflects a municipality’s degree of centrality. It is calculated by Statistics Norway based on travel time to workplaces and high-order service functions and the result is grouped in categories from 1 (most central) to 6 (least central) [22].

NORCAR can extract and prepare aggregated, anonymous results without further ethical approval. A NORCAR administrator (JKJ) extracted, recoded and aggregated the data on request from the main author (Norwegian Institute of Public Health, ref.nr HKR 19–0149).

### Preprocessing of data

We recoded the municipality where the event took place, into centrality category from 1 (most central) to 6 (least central) based upon data from Statistics Norway [22].

We calculated ambulance response intervals as the time interval between first call received at EMCC to the time when the first ambulance was at the patient’s location. For ambulance witnessed arrests, response interval was set to zero. We regarded negative or no values for response interval as missing values. We excluded cases with response intervals exceeding 120 min.

CPR duration was calculated as the time interval from when CPR started to the time CPR ended. If “Time CPR started” was missing, we did the following exchanges: 1. If cardiac arrest was witnessed by the ambulance, we used “Time of cardiac arrest” as “Time CPR started”. 2. If the first rhythm was registered as ventricular fibrillation (VF) or ventricular tachycardia (VT), we used “Time first defibrillation” as “Time CPR started”.

If “Time CPR ended” was missing, we did the following exchanges: 1. If the patient had ROSC, we used “Time sustained ROSC” as “Time CPR ended”. 2. If patient arrived at hospital with ongoing CPR, we used “Time arrived at hospital” as “Time CPR ended”. We regarded negative values and no values for CPR duration as missing values. We excluded cases with CPR duration of more than 120 min. The time limit on response interval and CPR duration enabled a valid data extraction from the registry.

### Data extraction and filtering

**Fig. 1:**
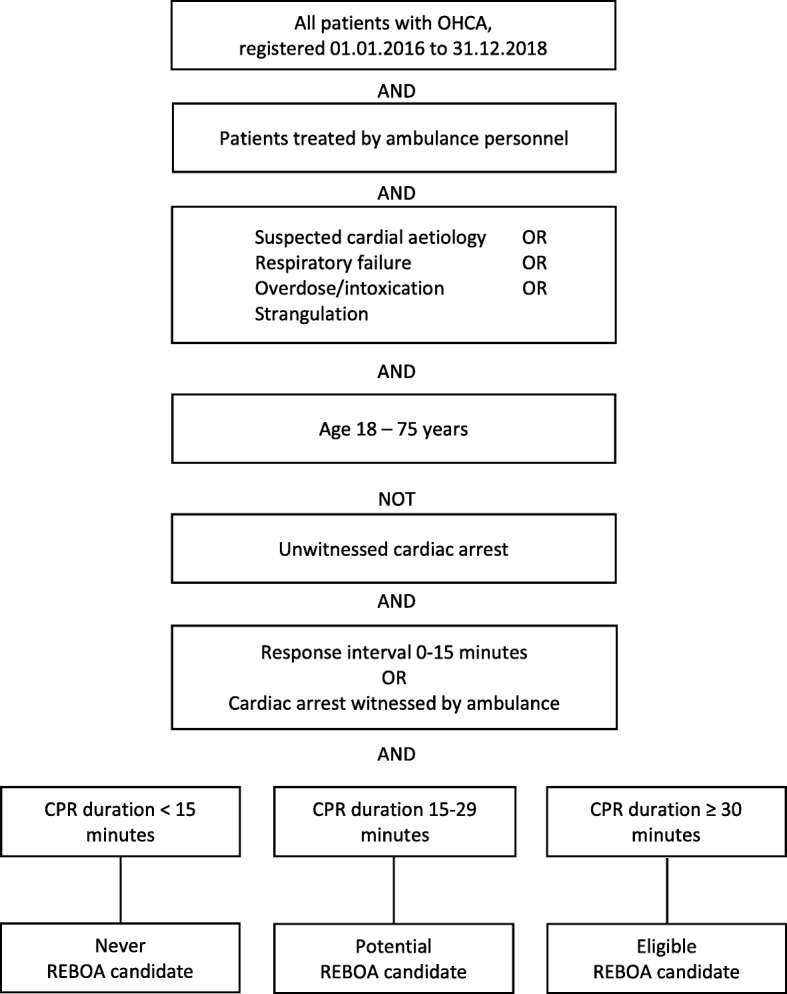
Data extraction from the Norwegian Cardiac Arrest Registry followed this flowchart. OHCA, out of hospital cardiac arrest. CPR, cardiopulmonary resuscitation. REBOA, resuscitative endovascular balloon occlusion of the aorta

We analyzed data in sub-groups as presented in the flowchart in Fig. 1. The data is split into three groups, based on duration of CPR; CPR duration less than 15 min (never eligible), CPR duration from 15 to 29 min (potentially eligible), and CPR duration longer than 30 min (eligible).

### Statistical analysis

Continuous variables are reported as median with interquartile range (IQR 1–3). Categorical variables are described as count and/or proportion (%). Statistical analyses are performed with SPSS (IBM Corp. Released 2017. IBM SPSS Statistics for Windows, Version 25.0. Armonk, NY: IBM Corp). Proportions are analyzed with Chi-squared tests and continuous measures with Kruskal-Wallis test. A *P* value of < 0.05 was regarded as statistically significant.

## Results

In the 3-year period 2016–2018 a total number of 10,488 OHCA were registered. Figure 1 and Table 1 depict results of stepwise data analyses.

**Table 1:** The characteristics of all patients with OHCA in the Norwegian Cardiac Arrest Registry from 2016 to 2018 (3 years). OHCA, out of hospital cardiac arrest. IQR, interquartile range. CPR, cardiopulmonary resuscitation

Characteristics	Missing data	Number of patients
All patients with OHCA (n)		**10,488**
Age (median, IQR)		69 (54–79)
Gender (% male)		66
Treated by ambulance personnel (n)		8339
Non traumatic arrests (n)		7551
Age 18–75 (n)	5	4596
Age (median, IQR)		62 (52–69)
Gender (% male)		71
Witnessed collapse (n)	106	2772
Treated by ambulance personnel within 15 min (n)	35	2241
CPR duration (n)	277	
< 15 min		716
15–29 min		528
> 30 min		**720**

We excluded 2149 patients that were not treated by ambulance personnel. Nine hundred seventeen cases were considered futile, 71 cases had do-not-resuscitate status, in 65 cases resuscitation was aborted due to pre-existing comorbidity, in 989 cases circulation was detected at ambulance arrival and in 107 cases no reason for not treating the patient was reported. Secondly, we filtered based on presumed cause of arrest and excluded 788 patients. The presumed cause was neurological in 168 cases, drowning in 128 cases, non-traumatic hemorrhage in 162 cases, hypothermia in 12 cases, fire/trauma in 293 cases and sudden infant death syndrome in 25 cases. After restricting age range and excluding non-witnessed cases, we grouped 2241 cases with response interval shorter than 15 min, into three groups based on CPR duration (Table 2).

**Table 2:** Characteristics and outcome in three subgroups of patients. Response interval and bystander CPR proportion are only calculated for non-ambulance witnessed arrest. Group differences in age and response interval are analyzed with Kruskal-Wallis test. Gender, presumed cause of arrest (cardiac vs non-cardiac) and bystander CPR started are analyzed with Chi-square test. Post-hoc tests are performed between specific groups and all *p*-values are corrected for multiple testing by Bonferroni correction. Only significant p-values are reported. CPR, cardiopulmonary resuscitation. IQR, interquartile range. VF/VT, ventricular fibrillation/ventricular tachycardia. PEA, pulseless electrical activity. ROSC, return of spontaneous circulation

	Group 1 CPR duration	Group 2 CPR duration	Group 3 CPR duration	Statistical tests
	**< 15 min**	**15–29 min**	**> 30 min**	
n	**716**	**528**	**720**	
Male, n (%)	544 (76)	358 (68)	522 (73)	*P* < 0.05 1 vs 2, *P* = 0.004
Age, median (IQR)	61 (52–69)	65 (55–70)	65 (56–70)	*P* < 0.01 1 vs 2, *P* < 0.001 1 vs 3, *P* < 0.001
Arrest witnessed by ambulance, n (%)	233 (33)	96 (18)	180 (25)	*P* < 0.001 1 vs 2, *P* < 0.001 1 vs 3, *P* = 0.006 2 vs 3, *P* = 0.01
Response time (min), median (IQR)	7 (5–10)	8 (6–11)	9 (7–12)	*P* < 0.01 1 vs 3, *P* < 0.001 2 vs 3, *P* < 0.001
CPR duration (min), median (IQR)	6 (3–11)	22 (19–26)	55 (35–55)	
Presumed cause, n (%)				*P* = 0.01
Cardiac	569 (80)	391 (74)	583 (81)	Post-hoc tests
Respiratory	89 (12)	86 (16)	101 (14)	non-significant
Overdose/intoxication	28 (4)	24 (5)	17 (2)	
Strangulation	30 (4)	27 (5)	19 (3)	
Bystander CPR, n (%)	422 (87)	353 (82)	437 (81)	*P* = 0.01 1 vs 2, *P* = 0.05 1 vs 3, *P* = 0.02
Initial rhythm, n (%)				
VF/VT	427 (60)	171 (32)	275 (38)	
PEA	121 (17)	126 (24)	163 (23)	
Asystole	105 (15)	214 (41)	251 (35)	
Unknown	63 (9)	17 (3)	31 (4)	
Centrality class, n (%)				
1 (most central)	204 (28)	120 (23)	127 (18)	
2	121 (17)	88 (17)	99 (14)	
3	161 (22)	134 (25)	181 (25)	
4	90 (13)	66 (13)	114 (16)	
5	44 (6)	38 (7)	85 (12)	
6 (least central)	11 (2)	6 (1)	28 (4)	
missing	85 (12)	66 (13)	86 (12)	
**Sustained ROSC, n (%)**	**609 (85)**	**251 (48)**	**132 (18)**	
**30-day survival, n (%)**	**459 (64)**	**102 (19)**	**52 (7)**	

**Fig. 2:**
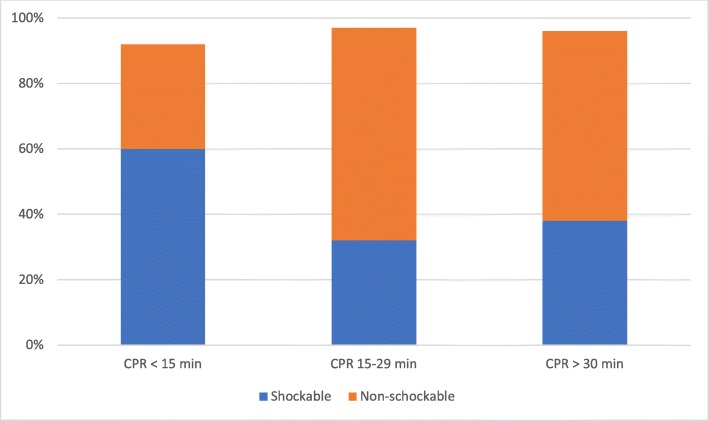
Proportions of initial rhythm in the three subgroups. Cases with missing initial rhythm is not shown. CPR, cardiopulmonary resuscitation

Table 2 shows that the three groups have minor differences in gender, age, response interval and presumed cause of arrest, with cardiac aetiology as the most frequent cause. There is a high proportion of bystander CPR (> 80%) before ambulance arrival in all groups. Figure 2 shows that there is a greater proportion of shockable initial rhythm in the group with shortest CPR duration.

**Fig. 3:**
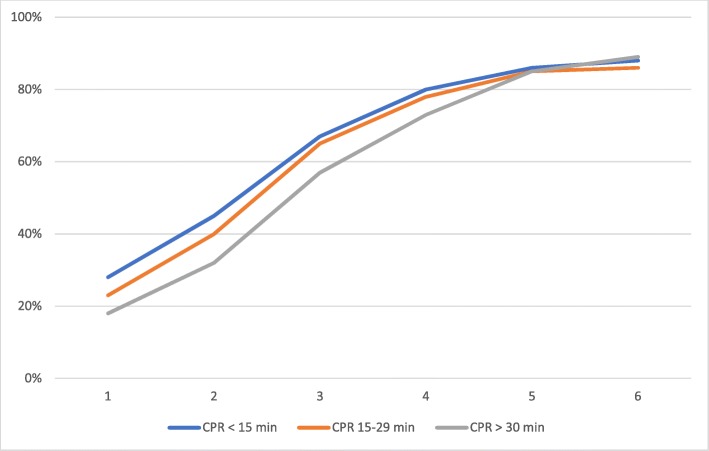
Cumulative distribution of centrality class in the three subgroups. Centrality class 1 is most central, class 6 is least central. CPR, cardiopulmonary resuscitation

Figure 3 show the distribution of centrality class in the three subgroups (class 1 being most central). In the group eligible for REBOA, most cases are from central areas, with only 16% of cases from the least central areas (class 5–6).

**Fig. 4:**
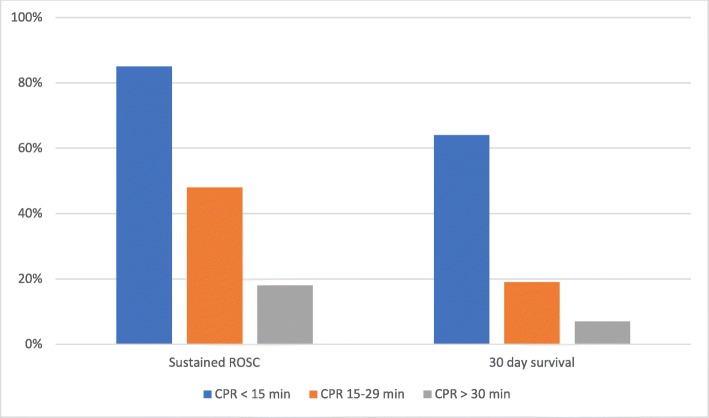
30-day survival and ROSC in the three subgroups. CPR, cardiopulmonary resuscitation. ROSC, return of spontaneous circulation

A distinct decrease in ROSC and 30-day survival in the three subgroups are shown in Fig. 4. In the group with CPR duration more than 15 min (eligible group), 18% of patient achieve ROSC and 7% survive for 30 days or more.

## Discussion

This is the first study to report the estimated number of potentially eligible patients for REBOA in OHCA. Our analysis demonstrates that over a three-year period 720 patients in Norway were potentially eligible. This corresponds to 240 patients per year or approximately 9% of the total number of ambulance treated OHCA. The patient group is predominately young with a median age of 65 years with a high potential for good long-term functionality.

The 30-day survival rate of the total OHCA patients in Norway in the period of observation is 14%, and 83% of these patients had good neurological outcome, measured as Cerebral Performance Category 1 or 2 [1], a scale often used to assess neurological outcome after cardiac arrest [23, 24]. Studies report that few OHCA patients overall have terminal illness and most have no or mild comorbidity [2, 25]. Pre-hospital healthcare providers are faced with the question of when to continue or terminate a resuscitation attempt, mostly without knowledge of any previous “do-not-resuscitate” decisions or absolute “termination-of-resuscitation”-rules. We commonly accept that the decision to stop resuscitation rely on the healthcare providers qualitative judgement [26]. The guidelines from the Norwegian Resuscitation Council recommend to continue CPR if there is persistent VF/VT, hypothermia, or as long as ethical/medical justifiable or signs of life [27]. Age is a factor that is easily obtained during resuscitation and is associated with increasing comorbidity and reduced life expectancy. Patient age has been associated with healthcare providers feelings of inappropriate CPR efforts [28]. However, one study report that older patients (> 70 years) may survive OHCA with favorable neurological outcome and that most have only mild or moderate comorbidity [29]. This clearly indicates that age alone is not a reliable argument to decide if the resuscitation is ethically or medically justifiable. Accordingly, we found it reasonable to include patients up to 75 years of age in this assessment of eligibility for pre-hospital REBOA in OHCA.

The differences between the groups (Table 2) reflects the selection process based on CPR duration where it is reasonable to expect characteristics associated with favorable prognosis to be more prevalent among those with shorter CPR duration. Further exploration of these associations with multi-variate regression analysis is beyond the scope of this study.

Survival rates declines even after few minutes of CPR, [30] and in an unwitnessed arrest it is impossible to establish length of no-flow time. Unwitnessed cardiac arrests and unknown time of cardiac arrest was accordingly excluded from the analyses.

Twenty percent of the Norwegian population lives in rural areas, mountainous and coastal remote regions with limited access by road [31]. In our study, 16% of eligible patients had OHCA in the most remote municipalities (class 5–6). In a sub-arctic climate, this can entail time-consuming response with weather-dependent flight conditions. The EMCC dispatch regular ambulances and 18 helicopter emergency medical services (HEMS) units that covers the whole population [32]. Ambulance response intervals are generally short in Norway with a national median of 9 min [1], but HEMS units may have longer response intervals depending on patient location, other current missions or weather conditions. Ambulance response intervals longer than 15 min were excluded to avoid the potential bias of early withdrawal of care due to perceived futility.

We assumed a 15 min response interval for HEMS arrival. This may be optimistic in many cases, but exact numbers for this interval remain unavailable in NORCAR. The pilot study [17] demonstrated that the REBOA procedure takes approximately 12 min to perform in a pre-hospital setting. In addition to HEMS response time and time to establish ACLS (airway management, mechanical chest compression machine and intravenous/intraosseous access on the upper body), we estimated that only patients receiving CPR for > 30 min are eligible. The ambulance response time and HEMS response time are arbitrarily set by the authors opinions.

In the group of patients with CPR duration of 15–29 min, one-half achieved ROSC (48%), and for the rest, CPR efforts were terminated for unknown reasons before 30 min. Some of these patients might be eligible for the REBOA procedure if the HEMS response interval were shorter, and if the procedure could be performed faster, or in parallel with other ACLS interventions.

The initial rhythm was VF/VT in 38% of the eligible cases. This is a higher proportion than in the general OHCA population, as expected and in accordance with the recommendations from the Norwegian Resuscitation Council. We chose to include non-shockable initial rhythms as possible inclusion criteria for pre-hospital REBOA. For this group, the results of conventional treatment are generally poor and new interventions that may improve aortic blood pressures could be especially beneficial. NORCAR do not have information about changes in cardiac rhythm during resuscitation, specifically, what cardiac rhythm presented at the possible time of REBOA inclusion.

A gap analysis compares current knowledge or practice to potential or desired performance. In a medical context a better phrase is *needs assessment,* where systematic analyses may identify changes in practice to optimize treatment. A description of a health problem allow optimal allocation of resources to improve the health in a population [33, 34]. It can be tempting to implement new and innovative interventions, but some of these might be resource intensive, carry potential hazards for patients, not properly tested for effect or they may divert focus from well-documented conventional treatment. It is therefore important to perform a needs assessment [33] before any new intervention is universally tested for effect on outcome. A needs assessment may provide the number of patients eligible for inclusion of a specific procedure, e.g. REBOA as an adjunct treatment in non-traumatic OHCA.

Pre-hospital REBOA procedure is shown to be feasible during ACLS, with preserved ACLS quality [17]. This was performed by a standard Norwegian HEMS crew, after completion of a structured training program [35]. The Norwegian HEMS include anaesthesiologists and are regularly part of the resuscitation team at OHCA [36]. This provides a pre-hospital competency in establishing central vascular lines using Seldinger technique [37]. Services without this competence in the field may not easily be able to perform a pre-hospital REBOA procedure. A structured educational program and regular procedural training is essential for procedural success. With 18 HEMS bases and approximately 240 eligible patients each year, it is not likely that all Norwegian HEMS bases should be trained in this procedure. Most patients are in areas with high centrality index and we therefore argue that only the most central HEMS bases should receive this comprehensive educational program [35].

### Limitations

This study has several limitations. First, it is mandatory to register OHCA to the registry, but some missing inclusion is nevertheless likely. One Swedish study report that as many as 25% of OHCA was not reported to the Swedish Cardiac Arrest Registry [38]. This is a possible cause of selection bias. Further, there are no data from resuscitations performed at nursing homes unless an EMCC was contacted and no data from out-of-hours primary health care centers unless an EMCC was contacted. These two groups normally constitute only a small number each year, and generally holds a poor prognosis. Second, this is a needs assessment solely based on one database review, thereby only providing an estimate of index cases. The lack of data on actual involvement of HEMS-crew in treatment and their response intervals necessitated some assumptions. However, the registry is nation-wide, and we would argue that because of the solidity of the data, the number of eligible patients is likely to be clinically accurate. Third, NORCAR is a live database and all local clerks can add or amend cases. Therefore, the data set may change slightly even for historical data, but expected changes are minimal. Fourth, 277 of the 2241 patients with possible inclusion, had missing data on CPR duration, this constitutes 12%. We decided not to perform multiple imputation [39] as by NORCAR experience the missing data is not missing at random. Fifth, we selected only cases with a suspected non-traumatic aetiology. Some of the patients in the “strangulation” category could be due to a high energy injury/strangulation and should therefore be deemed as a traumatic cardiac arrest. The suspected aetiology is as perceived by the health providers present on scene and may differ from results after autopsy, e.g. is the presumed cause overdose/intoxication (Table 2) which can be related to hypoxia rather than the intoxication itself. Autopsy after deaths outside hospitals are rare in Norway. Sixth, the result of our needs assessment is based on a Norwegian cardiac arrest registry and the Norwegian physician-manned HEMS structure. This may not be generalizable to other nations, with different population distribution or different policies and cultures for “end-of-resuscitation” decisions. Last, a major challenge in the pre-hospital setting is weather and light conditions, temperature and amount of space available around the patient, as well as availability of HEMS crew. This may impact on the number of potential candidates that are truly eligible for the procedure. This is well-known operational factor in pre-hospital care but is currently impossible to adjust for in this registry study.

## Conclusions

This analysis of data from a national registry constitutes a needs assessment of the REBOA procedure on OHCA. We found that each year approximately 240 patients, or 9% of the total ambulance treated OHCA in Norway may potentially be eligible for a pre-hospital REBOA procedure as an adjunct treatment to standard resuscitation. This patient cohort have 18% ROSC rate and 7% 30-day survival rate. Most of these patients are in central areas, with only 16% in non-central areas. This need assessment suggests that there is sufficient patient population in Norway to study REBOA as an adjunct treatment in non-traumatic OHCA. This is the first study to use national cardiac registry data to this purpose.

## Data Availability

The data that support the findings of this study are available from the Norwegian Cardiac Arrest Registry, but restrictions apply to the availability of these data, which were used under license for the current study, and so are not publicly available. Data are however available from the authors upon reasonable request and with permission of the Norwegian Cardiac Arrest Registry.

## Abbreviations

ACLS: Advanced cardiovascular life support
CA: Cardiac arrest
CPR: Cardiopulmonary resuscitation
EMCC: Emergency medical communications center
HEMS: Helicopter emergency medical service
NORCAR: Norwegian cardiac arrest registry
OHCA: Out of hospital cardiac arrest
REBOA: Resuscitative endovascular balloon occlusion of the aorta
ROSC: Return of spontaneous circulation
